# β-K_3_Fe(MoO_4_)_2_Mo_2_O_7_

**DOI:** 10.1107/S1600536814013087

**Published:** 2014-06-11

**Authors:** Amira Souilem, Mohamed Faouzi Zid, Ahmed Driss

**Affiliations:** a Laboratoire de Matériaux et Cristallochimie, Faculté des Sciences de Tunis, Université de Tunis ElManar, 2092 Manar II Tunis, Tunisia

## Abstract

The title compound, tripotassium iron(III) bis­(ortho­molyb­date) dimolybdate, was obtained by a solid-state reaction. The main structural building units are one FeO_6_ octa­hedron, two MoO_4_ tetra­hedra and one Mo_2_O_7_ dimolybdate group, all with point group symmetries *m*. These units are linked *via* corner-sharing to form ribbons parallel to [010]. The three K^+^ cations are located between the ribbons on mirror planes and have coordination numbers of 10 and 12. Two O atoms of one of the MoO_4_ tetra­hedra of the dimolybdate group are disordered over two positions in a 0.524 (11):0.476 (11) ratio. The structure of the title compound is compared briefly with that of Rb_3_FeMo_4_O_15_.

## Related literature   

For properties of related molybdates, see: Zhang *et al.* (2000 ▶); Muessig *et al.* (2003 ▶); Bramnik *et al.* (2003 ▶); Soares & Portela (2005 ▶); Bowker *et al.* (2008 ▶); Otko *et al.* (1978 ▶); Klimin *et al.* (2003 ▶); Jorge *et al.* (2004 ▶); Maczka *et al.* (2005 ▶). The closely related crystal structures of α-K_3_Fe(MoO_4_)_2_(Mo_2_O_7_) and Rb_3_Fe(MoO_4_)_2_Mo_2_O_7_ were determined by Maczka *et al.* (2009 ▶) and Khal’baeva *et al.* (2010 ▶), respectively. For bond-valence sums, see: Brown & Altermatt (1985 ▶).

## Experimental   

### 

#### Crystal data  

K_3_Fe(MoO_4_)_2_Mo_2_O_7_
*M* *_r_* = 796.91
Monoclinic, 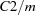

*a* = 32.873 (2) Å
*b* = 5.7137 (7) Å
*c* = 7.9177 (9) Å
β = 91.143 (8)°
*V* = 1486.9 (3) Å^3^
*Z* = 4
Mo *K*α radiation
μ = 5.15 mm^−1^
*T* = 298 K
0.36 × 0.22 × 0.18 mm

#### Data collection  

Enraf–Nonius CAD-4 diffractometer
Absorption correction: ψ scan (North *et al.*, 1968 ▶) *T*_min_ = 0.286, *T*_max_ = 0.488
3443 measured reflections
1789 independent reflections
1636 reflections with *I* > 2σ(*I*)
*R*_int_ = 0.028
2 standard reflections every 120 min intensity decay: 1.3%

#### Refinement  

*R*[*F* ^2^ > 2σ(*F* ^2^)] = 0.029
*wR*(*F* ^2^) = 0.076
*S* = 1.06
1789 reflections
138 parameters
Δρ_max_ = 1.22 e Å^−3^
Δρ_min_ = −1.57 e Å^−3^

### 

Data collection: *CAD-4 EXPRESS* (Duisenberg, 1992 ▶; Macíček & Yordanov, 1992 ▶); cell refinement: *CAD-4 EXPRESS*; data reduction: *XCAD4* (Harms & Wocadlo, 1995 ▶); program(s) used to solve structure: *SHELXS97* (Sheldrick, 2008 ▶); program(s) used to refine structure: *SHELXL97* (Sheldrick, 2008 ▶); molecular graphics: *DIAMOND* (Brandenburg & Putz, 1999 ▶); software used to prepare material for publication: *WinGX* (Farrugia, 2012 ▶).

## Supplementary Material

Crystal structure: contains datablock(s) I. DOI: 10.1107/S1600536814013087/wm5025sup1.cif

Structure factors: contains datablock(s) I. DOI: 10.1107/S1600536814013087/wm5025Isup2.hkl

CCDC reference: 1006924

Additional supporting information:  crystallographic information; 3D view; checkCIF report

## Figures and Tables

**Fig. 1.**
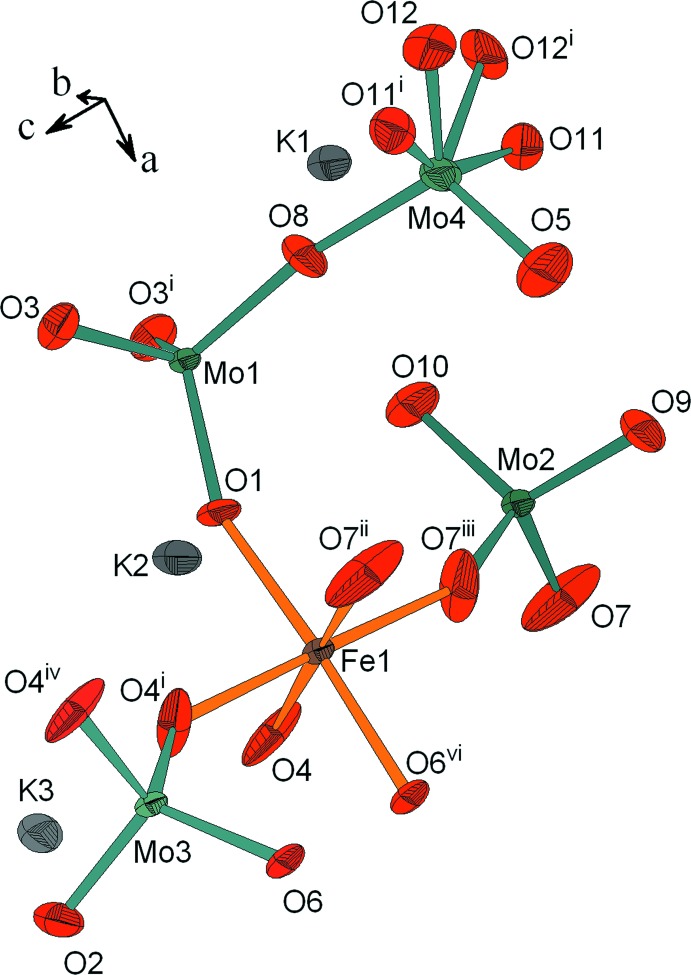
La structure de β-K3Fe(MoO4)2Mo2O7. Les éllipsoïdes ont été définis avec 50% de probabilité. [Symmetry codes: (i) x, -y+1, z; (ii) x, y, z-1; (iii) x, -y, z; (iv) x, y-1, z; (v) x, -y+1, z-1 ; (vi) -x+3/2, -y+1/2, -z].

**Fig. 2.**
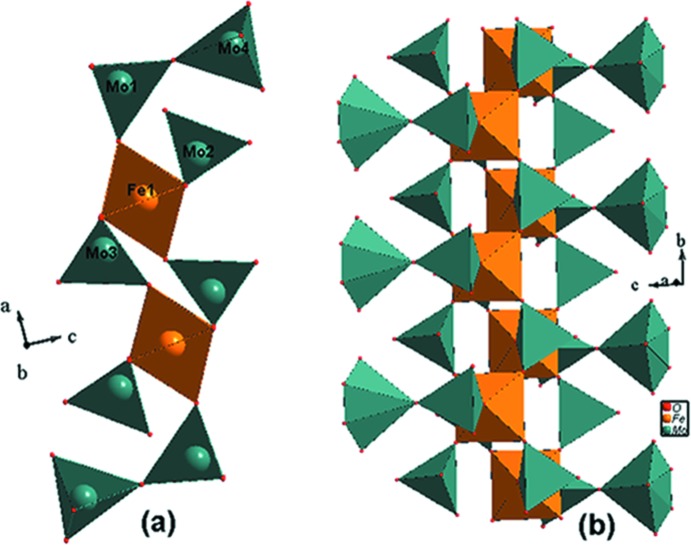
(a): Nouvelle unité centrosymétrique. (b): Vue, selon [010], d'un ruban dans β-K3Fe(MoO4)2Mo2O7,

**Fig. 3.**
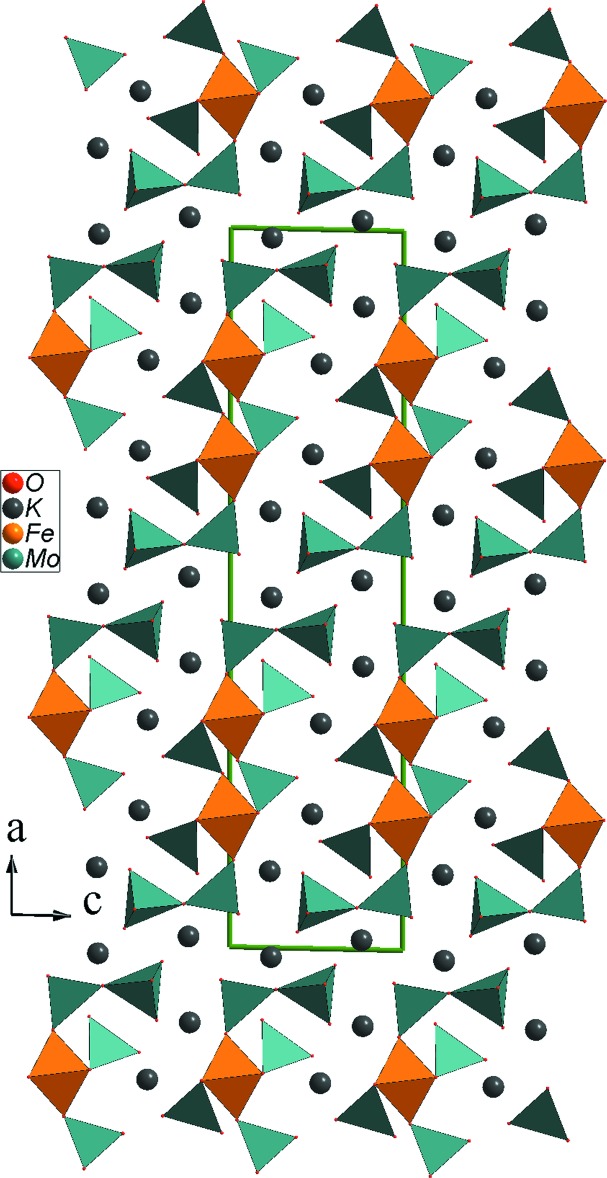
Projection de la structure de β-K3Fe(MoO4)2Mo2O7, selon b, montrant les espaces inter-rubans où résident les cations.

**Fig. 4.**
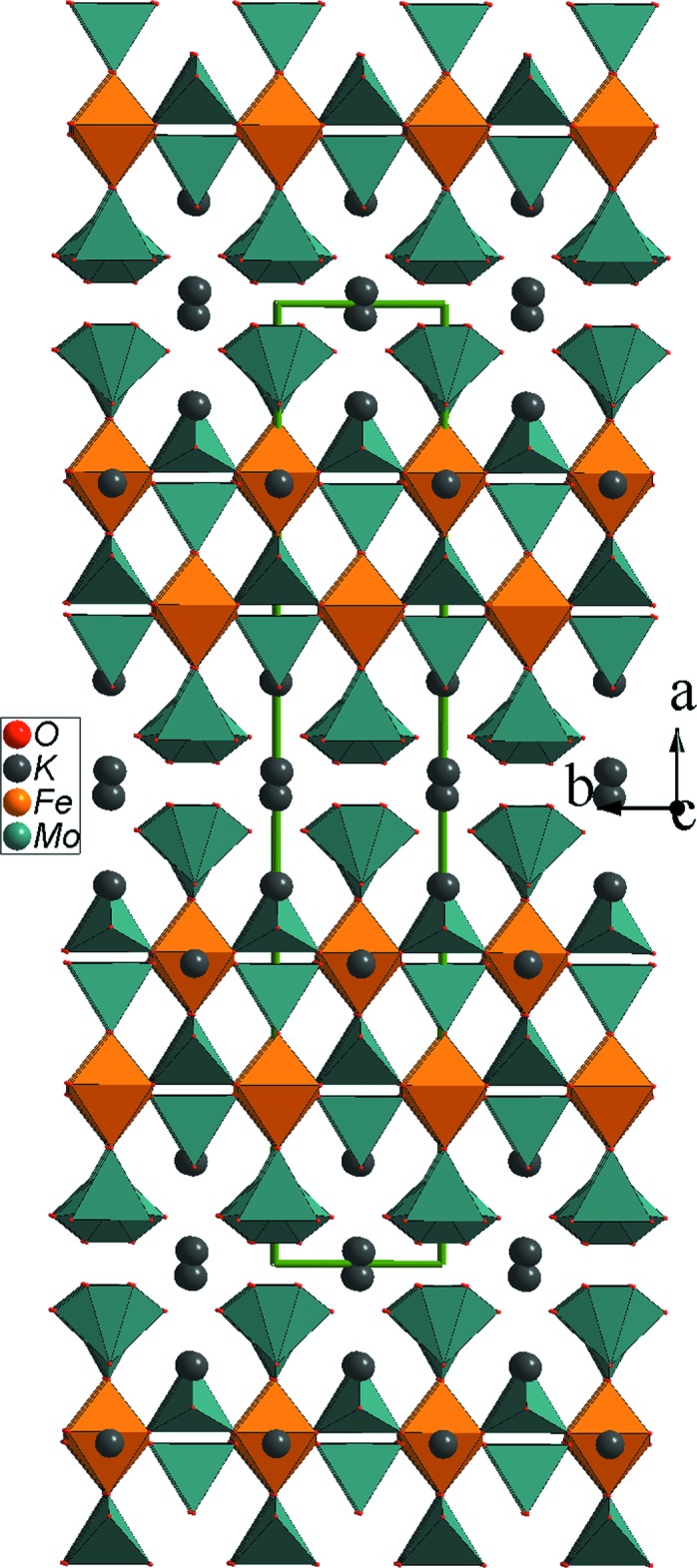
Projection de la structure de β-K3Fe(MoO4)2Mo2O7, selon c.

**Fig. 5.**
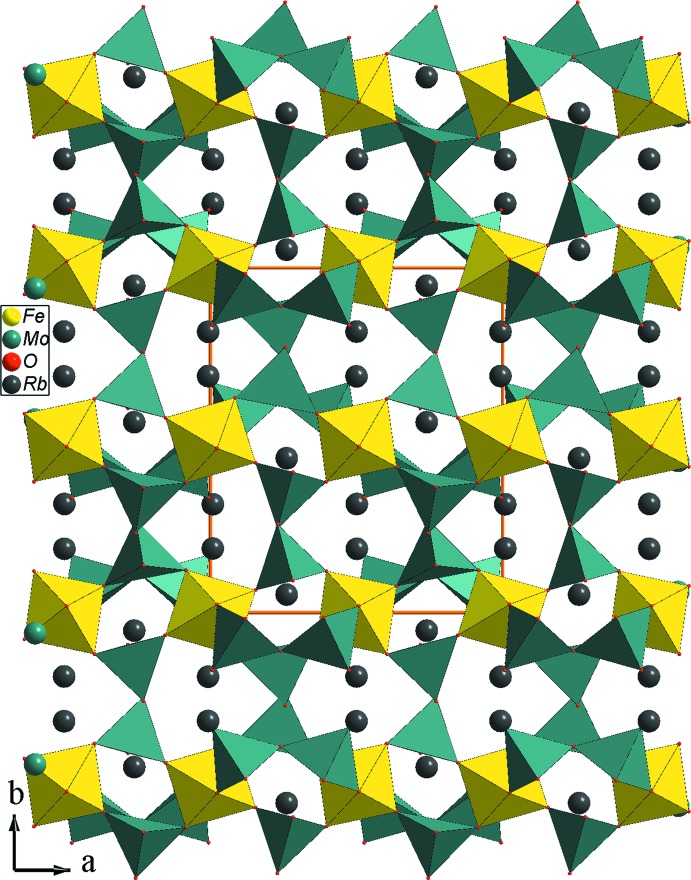
Projection de la structure de Rb3Fe(MoO4)2Mo2O7 selon c.

**Fig. 6.**
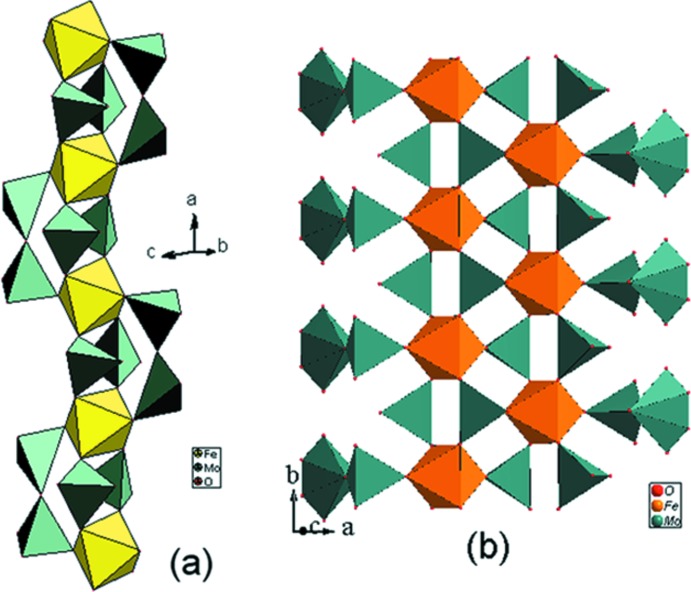
Représentation des rubans mettant en évidence les environnements des octaèdres FeO6 (a): Dans Rb3Fe(MoO4)2Mo2O7; (b): Dans β-K3Fe(MoO4)2Mo2O7.

## supplementary crystallographic information

### S1. Comment

L'étude des composés appartenant à la grande famille des molybdates de fer, métaux alcalins et pseudo-alcalins qui possèdent des propriétés structurelles, catalytiques, magnétiques et ferroélastiques intéressantes (Zhang *et al.*, 2000; Muessig *et al.*, 2003; Bramnik *et al.*, 2003; Soares & Portela, 2005; Bowker *et al.*, 2008; Otko *et al.*, 1978; Maczka *et al.*, 2005; Klimin *et al.*, 2003; Jorge *et al.*, 2004) nous a encouragés à explorer les systèmes molybdates de fer à cations monovalents. Une étude bibliographique a été effectuée sur la famille de composés de formulation *A*Fe(III)Mo_4_O_15_ où *A* est un cation monovalent: α-K_3_Fe(MoO_4_)_2_(Mo_2_O_7_) (Maczka *et al.*, 2009), Rb_3_FeMo_4_O_15_ (Khal'baeva *et al.*, 2010). En effet, le présent travail porte sur l'étude structurale d'un molybdate de formulation β-K_3_Fe(MoO_4_)_2_(Mo_2_O_7_) obtenu par voie sèche.

La structure de K_3_Fe(MoO_4_)_2_(Mo_2_O_7_) (forme-β) est de formule analogue à une autre phase déjà connue: K_3_Fe(MoO_4_)_2_(Mo_2_O_7_) (forme-α; Maczka *et al.*, 2009). Elles pésentent la même symétrie monoclinique.

La nouvelle variété β-K_3_Fe(MoO_4_)_2_(Mo_2_O_7_) est formée d'un octaèdre FeO_6_, de deux tétraèdres MoO_4_ et d'un groupement dimolybdate Mo_2_O_7_ (Fig. 1). Deux unités formulaires centrosymétriques se regroupent par partage de sommets donnant lieu à un dimère de formulation (FeO_6_)_2_(Mo_4_)_4_(Mo_2_O_7_)_2_ (Fig. 2*a*). Ces derniers se connectent moyennant des ponts mixtes de type Mo–O–Fe pour donner des rubans disposés selon [010] (Fig. 2*b*). Il en résulte une charpente unidimensionnelle possédant des espaces inter-rubans où résident les cations monovalents K^+^ (Figs. 3 et 4).

L'examen des facteurs géométriques dans la structure montre qu'ils sont en bon accord avec ceux rencontrés dans la littérature (Maczka *et al.*, 2009; Khal'baeva *et al.*, 2010).

D'autre part, le calcul des valences de liaisons (BVS), utilisant la formule empirique de Brown (Brown & Altermatt, 1985), conduit aux valeurs des charges des cations suivants: Fe1 (3,03), Mo1 (6,03), Mo2 (6,13), Mo3 (5,94), Mo4 (6,03), K1 (0,61), K2 (0,71) et K3 (0,59).

Une comparaison de la structure du composé étudié β-K_3_Fe(MoO_4_)_2_(Mo_2_O_7_) avec celle de Rb_3_Fe(MoO_4_)_2_Mo_2_O_7_ (Khal'baeva *et al.*, 2010) montre une différence nette dans la disposition des polyèdres et en particulier des groupements dimolybdates Mo_2_O_7_ dans la charpente anionique (Fig. 5).

En effet, on remarque que pour le composé au rubidium, cette disposition selon la direction des rubans, permet à chaque groupement dimolybdate Mo_2_O_7_ de relier par partage de sommets deux octaèdres FeO_6_ différents appartenant au même ruban (Fig. 6*a*). Par contre dans le composé étudié chaque octaèdre FeO_6_ partage un sommet avec un seul groupement dimolybdate Mo_2_O_7_, les cinq autres sommets sont mis en commun avec respectivement cinq tétraèdres MoO_4_ différents (Fig. 6*b*). Nous pouvant signaler que le second tétraèdre terminal du groupement Mo_2_O_7_ présente un atome d'oxygène délocalisé sur deux positions proches O11 et O12 donnant une forme pyramidale au tétraèdre (Fig. 6*b*).

### S2. Experimental

La synthèse de K_3_FeMo_4_O_15_ a été effectuée par réaction à l'état solide, à partir de carbonate de potassium (Prolabo, 60109), de nitrate de fer (Fluka 44949) et de molybdate d'ammonium (Fluka, 69858) dans un rapport molaire K:Fe:Mo égal à 3:1:6 respectivement. Après un broyage poussé dans un mortier en agate, le mélange est placé dans un creuset en porcelaine, puis porté dans un premier temps à une température de 673 K pendant 4 heures, en vue d'éliminer les produits volatils. Un second traitement thermique a été effectué à la température de synthèse, 1126 K pendant 24 heures. Le résidu final est ensuite ramené à un refroidissement lent de 5 K/12 h jusqu'à 1043 K, suivi d'un autre plus rapide (50 K/24 h) jusqu'à la température ambiante. Des cristaux de couleur verdâtre sont séparés à l'eau chaude.

### S3. Refinement

L'examen de la différence de fourier final à révélé la présence d'un pic proche de la position de l'atome d'oxygène O11. L'insertion de ce pic O12 et l'affinement des taux d'occupation [0.524 (11) et 0.476 (11) pour respectivement O11 et O12] conduit à des ellipsoïdes bien définis. De plus, les densités d'électrons maximum et minimum restants dans la Fourier-différence sont acceptables et sont situées respectivement à 0,43 Å de Mo4 et à 0,92 Å de Mo4.

### Crystal data

**Table:**

K_3_Fe(MoO_4_)_2_Mo_2_O_7_	*F*(000) = 1484
*M**_r_* = 796.91	*D*_x_ = 3.560 Mg m^−^^3^
Monoclinic, *C*2/*m*	Mo *K*α radiation, λ = 0.71073 Å
Hall symbol: -C 2y	Cell parameters from 25 reflections
*a* = 32.873 (2) Å	θ = 11–15°
*b* = 5.7137 (7) Å	µ = 5.15 mm^−^^1^
*c* = 7.9177 (9) Å	*T* = 298 K
β = 91.143 (8)°	Prism, green
*V* = 1486.9 (3) Å^3^	0.36 × 0.22 × 0.18 mm
*Z* = 4	

### Data collection

**Table:**

Enraf–Nonius CAD-4 diffractometer	1636 reflections with *I* > 2σ(*I*)
Radiation source: fine-focus sealed tube	*R*_int_ = 0.028
Graphite monochromator	θ_max_ = 27.0°, θ_min_ = 2.5°
ω/2θ scans	*h* = −41→41
Absorption correction: ψ scan (North *et al.*, 1968)	*k* = −1→7
*T*_min_ = 0.286, *T*_max_ = 0.488	*l* = −10→5
3443 measured reflections	2 standard reflections every 120 min
1789 independent reflections	intensity decay: 1.3%

### Refinement

**Table:**

Refinement on *F*^2^	Primary atom site location: structure-invariant direct methods
Least-squares matrix: full	Secondary atom site location: difference Fourier map
*R*[*F*^2^ > 2σ(*F*^2^)] = 0.029	*w* = 1/[σ^2^(*F*_o_^2^) + (0.0306*P*)^2^ + 20.6944*P*] where *P* = (*F*_o_^2^ + 2*F*_c_^2^)/3
*wR*(*F*^2^) = 0.076	(Δ/σ)_max_ = 0.001
*S* = 1.06	Δρ_max_ = 1.22 e Å^−^^3^
1789 reflections	Δρ_min_ = −1.57 e Å^−^^3^
138 parameters	Extinction correction: *SHELXL97* (Sheldrick, 2008), Fc^*^=kFc[1+0.001xFc^2^λ^3^/sin(2θ)]^-1/4^
0 restraints	Extinction coefficient: 0.00379 (16)

### Special details

**Table:**

Geometry. All e.s.d.'s (except the e.s.d. in the dihedral angle between two l.s. planes) are estimated using the full covariance matrix. The cell e.s.d.'s are taken into account individually in the estimation of e.s.d.'s in distances, angles and torsion angles; correlations between e.s.d.'s in cell parameters are only used when they are defined by crystal symmetry. An approximate (isotropic) treatment of cell e.s.d.'s is used for estimating e.s.d.'s involving l.s. planes.
Refinement. Refinement of *F*^2^ against ALL reflections. The weighted *R*-factor *wR* and goodness of fit *S* are based on *F*^2^, conventional *R*-factors *R* are based on *F*, with *F* set to zero for negative *F*^2^. The threshold expression of *F*^2^ > σ(*F*^2^) is used only for calculating *R*-factors(gt) *etc*. and is not relevant to the choice of reflections for refinement. *R*-factors based on *F*^2^ are statistically about twice as large as those based on *F*, and *R*- factors based on ALL data will be even larger.

### Fractional atomic coordinates and isotropic or equivalent isotropic displacement parameters (Å^2^)

**Table:**

	*x*	*y*	*z*	*U*_iso_*/*U*_eq_	Occ. (<1)
Mo1	0.568576 (15)	0.5000	−0.03610 (7)	0.01354 (16)	
Mo2	0.649834 (16)	0.0000	0.73776 (7)	0.01577 (16)	
Mo3	0.718491 (15)	0.0000	0.18432 (7)	0.01355 (16)	
Mo4	0.55700 (2)	0.5000	0.49609 (8)	0.0375 (2)	
Fe1	0.68040 (3)	0.5000	−0.01846 (11)	0.0118 (2)	
K1	0.51309 (5)	0.0000	0.7616 (2)	0.0253 (3)	
K2	0.60638 (5)	0.0000	0.2307 (2)	0.0340 (4)	
K3	0.68689 (5)	0.5000	0.4756 (2)	0.0361 (5)	
O1	0.62085 (14)	0.5000	0.0211 (7)	0.0257 (11)	
O2	0.73729 (17)	0.0000	0.3855 (7)	0.0373 (15)	
O3	0.54601 (11)	0.7403 (7)	0.0504 (5)	0.0277 (8)	
O4	0.68711 (12)	0.7469 (9)	0.1610 (6)	0.0459 (13)	
O5	0.60430 (19)	0.5000	0.4216 (9)	0.053 (2)	
O6	0.75919 (14)	0.0000	0.0434 (6)	0.0223 (11)	
O7	0.67523 (14)	0.2575 (11)	0.8080 (7)	0.0669 (19)	
O8	0.55792 (18)	0.5000	0.7337 (6)	0.0329 (13)	
O9	0.64948 (18)	0.0000	0.5221 (7)	0.0346 (14)	
O10	0.60110 (17)	0.0000	0.8120 (8)	0.0396 (15)	
O11	0.5491 (2)	0.1696 (14)	0.4498 (9)	0.031 (2)	0.524 (11)
O12	0.5224 (3)	0.3608 (15)	0.3916 (10)	0.034 (3)	0.476 (11)

### Atomic displacement parameters (Å^2^)

**Table:**

	*U*^11^	*U*^22^	*U*^33^	*U*^12^	*U*^13^	*U*^23^
Mo1	0.0084 (3)	0.0180 (3)	0.0142 (3)	0.000	−0.00063 (19)	0.000
Mo2	0.0111 (3)	0.0210 (3)	0.0151 (3)	0.000	−0.00068 (19)	0.000
Mo3	0.0080 (2)	0.0163 (3)	0.0164 (3)	0.000	−0.00035 (19)	0.000
Mo4	0.0169 (3)	0.0820 (6)	0.0137 (3)	0.000	0.0003 (2)	0.000
Fe1	0.0092 (4)	0.0101 (4)	0.0160 (4)	0.000	−0.0006 (3)	0.000
K1	0.0184 (7)	0.0324 (9)	0.0250 (8)	0.000	−0.0022 (6)	0.000
K2	0.0195 (7)	0.0563 (12)	0.0260 (8)	0.000	−0.0046 (6)	0.000
K3	0.0226 (8)	0.0665 (14)	0.0191 (8)	0.000	−0.0004 (6)	0.000
O1	0.010 (2)	0.042 (3)	0.025 (3)	0.000	−0.0025 (19)	0.000
O2	0.023 (3)	0.065 (4)	0.023 (3)	0.000	−0.008 (2)	0.000
O3	0.0224 (17)	0.0242 (18)	0.037 (2)	0.0016 (16)	0.0069 (15)	−0.0049 (17)
O4	0.031 (2)	0.051 (3)	0.058 (3)	−0.029 (2)	0.0281 (19)	−0.040 (2)
O5	0.027 (3)	0.085 (6)	0.048 (4)	0.000	0.011 (3)	0.000
O6	0.009 (2)	0.032 (3)	0.026 (3)	0.000	0.0045 (18)	0.000
O7	0.038 (2)	0.087 (4)	0.077 (4)	−0.036 (3)	0.026 (2)	−0.068 (3)
O8	0.035 (3)	0.048 (4)	0.015 (2)	0.000	−0.006 (2)	0.000
O9	0.033 (3)	0.056 (4)	0.015 (2)	0.000	−0.004 (2)	0.000
O10	0.018 (3)	0.056 (4)	0.045 (4)	0.000	0.005 (2)	0.000
O11	0.031 (4)	0.036 (4)	0.025 (4)	0.005 (3)	0.002 (3)	−0.002 (3)
O12	0.036 (5)	0.033 (5)	0.031 (4)	−0.017 (4)	−0.003 (3)	−0.015 (4)

### Geometric parameters (Å, º)

**Table:**

Mo1—O3	1.710 (4)	K1—O3^x^	2.882 (4)
Mo1—O3^i^	1.710 (4)	K1—O10	2.913 (6)
Mo1—O1	1.768 (5)	K1—O3^xi^	2.916 (4)
Mo1—O8^ii^	1.849 (5)	K1—O3^xii^	2.916 (4)
Mo2—O9	1.708 (5)	K1—O11^iii^	2.924 (7)
Mo2—O10	1.717 (6)	K1—O11	2.924 (7)
Mo2—O7	1.776 (5)	K1—O8^iv^	3.224 (3)
Mo2—O7^iii^	1.776 (5)	K2—O9	2.683 (6)
Mo3—O2	1.697 (5)	K2—O11	2.762 (7)
Mo3—O6	1.759 (5)	K2—O11^iii^	2.762 (7)
Mo3—O4^iv^	1.784 (4)	K2—O3^iv^	2.841 (4)
Mo3—O4^i^	1.784 (4)	K2—O3^i^	2.841 (4)
Mo4—O12	1.605 (7)	K2—O4^i^	3.082 (5)
Mo4—O12^i^	1.605 (7)	K2—O4^iv^	3.082 (5)
Mo4—O5	1.674 (6)	K2—O5	3.233 (3)
Mo4—O8	1.881 (5)	K2—O5^iv^	3.233 (3)
Mo4—O11	1.940 (8)	K2—O10^ii^	3.317 (7)
Mo4—O11^i^	1.940 (8)	K2—O1^iv^	3.343 (3)
Fe1—O7^ii^	1.956 (4)	K2—O1	3.343 (3)
Fe1—O7^v^	1.956 (4)	K3—O2^xiii^	2.704 (6)
Fe1—O1	1.989 (5)	K3—O5	2.740 (7)
Fe1—O6^vi^	2.000 (5)	K3—O4^i^	2.863 (5)
Fe1—O4	2.011 (4)	K3—O4	2.863 (5)
Fe1—O4^i^	2.011 (4)	K3—O7^i^	3.005 (7)
K1—O12^vii^	2.650 (8)	K3—O7	3.005 (7)
K1—O12^viii^	2.650 (8)	K3—O9	3.135 (3)
K1—O11^vii^	2.789 (8)	K3—O9^xiv^	3.135 (3)
K1—O11^viii^	2.789 (8)	K3—O2^xiv^	3.386 (3)
K1—O3^ix^	2.882 (4)	K3—O2	3.386 (3)
			
O3—Mo1—O3^i^	106.8 (3)	O12—Mo4—O11	47.9 (4)
O3—Mo1—O1	108.94 (15)	O12^i^—Mo4—O11	107.1 (4)
O3^i^—Mo1—O1	108.94 (15)	O5—Mo4—O11	93.2 (2)
O3—Mo1—O8^ii^	108.63 (16)	O8—Mo4—O11	100.9 (2)
O3^i^—Mo1—O8^ii^	108.63 (16)	O12—Mo4—O11^i^	107.1 (4)
O1—Mo1—O8^ii^	114.6 (2)	O12^i^—Mo4—O11^i^	47.9 (4)
O9—Mo2—O10	110.8 (3)	O5—Mo4—O11^i^	93.2 (2)
O9—Mo2—O7	107.9 (2)	O8—Mo4—O11^i^	100.9 (2)
O10—Mo2—O7	109.20 (18)	O11—Mo4—O11^i^	153.4 (4)
O9—Mo2—O7^iii^	107.9 (2)	O7^ii^—Fe1—O7^v^	90.2 (4)
O10—Mo2—O7^iii^	109.20 (18)	O7^ii^—Fe1—O1	92.22 (17)
O7—Mo2—O7^iii^	111.9 (4)	O7^v^—Fe1—O1	92.22 (17)
O2—Mo3—O6	109.2 (3)	O7^ii^—Fe1—O6^vi^	90.18 (17)
O2—Mo3—O4^iv^	107.2 (2)	O7^v^—Fe1—O6^vi^	90.18 (17)
O6—Mo3—O4^iv^	112.33 (14)	O1—Fe1—O6^vi^	176.6 (2)
O2—Mo3—O4^i^	107.2 (2)	O7^ii^—Fe1—O4	178.6 (2)
O6—Mo3—O4^i^	112.33 (14)	O7^v^—Fe1—O4	90.4 (3)
O4^iv^—Mo3—O4^i^	108.3 (3)	O1—Fe1—O4	89.03 (15)
O12—Mo4—O12^i^	59.4 (7)	O6^vi^—Fe1—O4	88.54 (16)
O12—Mo4—O5	118.2 (4)	O7^ii^—Fe1—O4^i^	90.4 (3)
O12^i^—Mo4—O5	118.2 (4)	O7^v^—Fe1—O4^i^	178.6 (2)
O12—Mo4—O8	120.8 (3)	O1—Fe1—O4^i^	89.03 (15)
O12^i^—Mo4—O8	120.8 (3)	O6^vi^—Fe1—O4^i^	88.54 (16)
O5—Mo4—O8	110.9 (3)	O4—Fe1—O4^i^	89.1 (3)

Symmetry codes: (i) *x*, −*y*+1, *z*; (ii) *x*, *y*, *z*−1; (iii) *x*, −*y*, *z*; (iv) *x*, *y*−1, *z*; (v) *x*, −*y*+1, *z*−1; (vi) −*x*+3/2, −*y*+1/2, −*z*; (vii) −*x*+1, −*y*, −*z*+1; (viii) −*x*+1, *y*, −*z*+1; (ix) −*x*+1, *y*−1, −*z*+1; (x) −*x*+1, −*y*+1, −*z*+1; (xi) *x*, −*y*+1, *z*+1; (xii) *x*, *y*−1, *z*+1; (xiii) −*x*+3/2, −*y*+1/2, −*z*+1; (xiv) *x*, *y*+1, *z*.
